# Change in antihypertensive drug prescribing after guideline implementation: a controlled before and after study

**DOI:** 10.1186/1471-2296-12-87

**Published:** 2011-08-17

**Authors:** Raija Sipilä, Arja Helin-Salmivaara, Maarit Jaana Korhonen, Eeva Ketola

**Affiliations:** 1 Current Care, Finnish Medical Society Duodecim, POB 713, 00100 Helsinki, Finland; 2 Department of Pharmacology, Drug Development and Therapeutics, University of Turku, 20014 Turku, Finland; 3 Unit of General Practice, Hospital District of Helsinki and Uusimaa, POB 705, FI-00029 Helsinki, Finland; 4 Department of Pharmacology, Drug Development and Therapeutics, University of Turku, FI-20014 Turku, Finland; 5 Institute of Public Health and Clinical Nutrition, Faculty of Health Sciences, University of Eastern Finland, POB 1627, FI-70211 Kuopio, Finland; 6 Helsinki City Health Department, POB 6000, FI-00099 City of Helsinki, Finland

## Abstract

**Background:**

Antihypertensive drug choices and treatment levels are not in accordance with the existing guidelines. We aimed to assess the impact of a guideline implementation intervention on antihypertensive drug prescribing.

**Methods:**

In this controlled before and after study, the effects of a multifaceted (education, audit and feedback, local care pathway) quality programme was evaluated. The intervention was carried out in a health centre between 2002 and 2003. From each health care unit (n = 31), a doctor-nurse pair was trained to act as peer facilitators in the intervention.

All antihypertensive drugs prescribed by 25 facilitator general practitioners (intervention GPs) and 53 control GPs were retrieved from the nationwide Prescription Register for three-month periods in 2001 and 2003. The proportions of patients receiving specific antihypertensive drugs and multiple antihypertensive drugs were measured before and after the intervention for three subgroups of hypertension patients: hypertension only, with coronary heart disease, and with diabetes.

**Results:**

In all subgroups, the use of multiple concurrent medications increased. For intervention patients with hypertension only, the odds ratio (OR) was 1.12 (95% CI 0.99, 1.25; p = 0.06) and for controls 1.13 (1.05, 1.21; p = 0.002). We observed no statistically significant differences in the change in the prescribing of specific antihypertensive agents between the intervention and control groups. The use of agents acting on the renin-angiotensin-aldosterone system increased in all subgroups (hypertension only intervention patients OR 1.19 (1.06, 1.34; p = 0.004) and controls OR 1.24 (1.15, 1.34; p < 0.0001).

**Conclusions:**

A multifaceted guideline implementation intervention does not necessarily lead to significant changes in prescribing performance. Rigorous planning of the interventions and quality projects and their evaluation are essential.

## Background

The association between high blood pressure and the risk of cardiovascular disease (CVD), such as stroke, is well-established [1-3]. Among the Finnish working age population, the estimated prevalence of hypertension was almost 50% in 1997, after which blood pressure levels declined until 2002 [4-7]. During the last decade, the use of antihypertensive drugs has continuously increased [8]. However, as in other European countries, only around every third hypertensive Finn achieves the recommended blood pressure level (< 140/90 mmHg) [4,9-12]. Furthermore, antihypertensive drug therapy is not in line with the current guidelines [9,13,14]; in Finland in particular the percentage of patients using beta-blocking agents is high irrespective of the type of co-morbidities [9]. In Finland, national evidence-based guidelines have been developed and published since 1994 in the Current Care Guideline Programme for both primary and secondary care, under the auspices of the Finnish Medical Society Duodecim.

In order to facilitate the adaptation of new research evidence, experts search, evaluate, and gather research findings into clinical guidelines. The mere act of publishing a guideline, unaccompanied by any other actions, is considered guideline dissemination. The next step towards proper implementation is distribution through various channels. Distributing educational material improves the care process by a median of 8% [15]. However 'real-life implementation' based on several, often simultaneous activities remains challenging. Single interventions typically have a small to moderate effect, with reminders proven to be the most effective (mean improvement in process of care 14%). Two Cochrane reviews show that outreach visits, as well as audit and feedback, exert a small, consistently positive impact on changes in prescription practices [16,17]. The more personalised the prescription intervention in a primary care setting is, the more effective it is thought to be [18,19].

One systematic guideline implementation study aiming at improving antihypertensive drug prescribing was conducted in Norway in 2002 [20-23]. This multifaceted intervention (educational outreach visits, audit and feedback, reminders, and risk assessment tools) was intended to increase the prescribing of thiazides as the first-line drugs for newly treated patients with hypertension. Compared with a control group exposed to passive dissemination, the prescription of thiazides increased significantly in the intervention group; however, intervention costs were the equivalent of twice the savings gained during the one-year follow-up.

We conducted a multifaceted quality improvement programme (education, audit and feedback, local care pathway and information) in Finland's primary care setting, aimed at implementing the existing guidelines and improving the clinical process in the prevention and care of cardiovascular diseases. We have described the intervention and the results concerning the process measures earlier [24,25]. Now we analyse the detailed effects of the intervention; the impact of the comprehensive implementation of the hypertension guidelines on the prescription of antihypertensive drugs in a controlled before and after design.

## Methods

### Setting

During the study period of 2002-2003, primary health care in Finland was mostly provided in municipal health centres run by salaried personnel. Practices were arranged either through the list system or the conventional system, whereby appointments could be arranged with any doctor working in a health centre. Of Finland's total population of 5.2 million in 2001 66% used public primary care services at least once [26]. Occupational health care and the private sector complemented primary health care services.

### Intervention

The Helsinki Prevention Programme was carried out in 2002-2003 at the Helsinki Health Centre, which covers population of 0.56 million [24]. The programme was aimed at local implementation of the national guidelines related to the prevention of, and care for, CVDs (hypertension, dyslipidaemia, obesity, diabetes and smoking). From each of the city's health care units (n = 31), a voluntary physician-nurse pair was recruited. They were trained as local experts in the prevention of CVDs and to act as intrinsic peer facilitators in guideline implementation. The two-year facilitator intervention consisted of 16 educational sessions (lectures, workshops, patient cases, and role modelling) and 12 distance learning tasks (auditing processes, self-audits, planning preventive activities, and educations for co-workers). The intervention has been described in detail earlier [24]. During the time of the intervention, patients were listed according to the place of residence.

**Figure 1:**
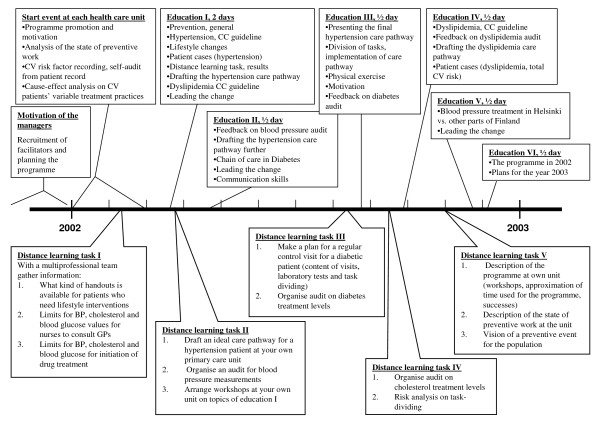
**Description of the intervention during the year 2002**. Abbreviations: CV, cardiovascular; CC, Current Care; GP, general practitioner; BP, blood pressure.

During the first year of the programme, the implementation focused on the hypertension guideline (Figure 1). The facilitators tailored a local care pathway for hypertension patients (a flowchart on the basis of the Current Care Guidelines) and further implemented the care pathway in interactive workshops in their own health care units [25]. In the flowchart, the diagnosis of hypertension, examinations and follow-up schema according to both total cardiovascular risk and blood pressure limits were described. The responsibilities of different health professionals and organisations were also described and the tasks were divided. Recommendations for antihypertensive medication (Table 1) were discussed in lectures and workshops; patient case reports were used to facilitate these discussions. Three core messages of the guideline were emphasised: 1) according to research evidence all four main antihypertensive drug groups are considered equally effective in lowering blood pressure, 2) drug choice should be based on the severity of hypertension, concomitant complications, the patient's other cardiovascular risk factors and co-morbidities, and 3) over half of all hypertension patients need multiple concurrent medications to reach treatment targets [27]. Furthermore, the financial aspects of the various drug choices were discussed.

**Table 1:** Recommendations for antihypertensive drug choices according to co-morbidities, the Current Care Hypertension guideline 2002 [27]

	First line medication	Second choice	Special considerations
**Hypertension**	Thiazide diuretics ACEI Beta-blocking agents	ARB	Diuretics or CCBs if isolated systolic blood pressure

**Diabetes**	ACEI Diuretics Beta-blocking agents CCBs		

**CHD**	Beta-blocking agents	Diuretics CCBs	

**Heart failure**	ACEI Diuretics	Beta-blocking agents ARB	

**Arrhythmia**	Beta-blocking agents CCBs		Depends on the type of arrhythmia

**Dyslipidaemia**			Do not have significance to the drug choice

**Asthma or COPD**	Diuretics CCBs ACEI		

Abbreviations: ACEI, angiotensin-converting enzyme inhibitor; ARB, angiotensin II receptor inhibitor; CCBs, calcium channel blockers; CHD, coronary heart disease; COPD, chronic obstructive pulmonary disease.

### Participating doctors and patients

Those 25 facilitator doctors who gave permission for data collection related to their prescriptions are referred to as intervention general practitioners (GPs). Voluntary controls (referred to as control GPs) were recruited from two large cities (Kuopio with the conventional and Turku with the list system). Two contact persons performed the recruitment, and 53 GPs gave permission (Kuopio n = 31 and Turku n = 22). At baseline, all participating physicians completed a questionnaire, giving background information and their self-rated motivation to treat patients in accordance with the Current Care Guidelines (based on the Finnish school grading system of 4-10). In this report, patients who were dispensed antihypertensive drugs during the study period in 2001 or 2003, as prescribed by the intervention or control GPs, are referred to as intervention and control patients for the respective year.

### Data source

The data on prescriptions was drawn from the National Prescription Register, managed by the Social Insurance Institution (SII). The register includes data on reimbursed medicines, and the patient's date of birth, gender and residential area [8,28]. In the register, the dispensed drugs are identifiable by the ATC classification code [29]. Each person can be identified through a unique personal ID and each doctor has a personal code. The register does not contain information on the purpose of the medication.

Reimbursement is divided into Basic and Special Refunds. Drugs with an approved reasonable wholesale price, including all antihypertensive drugs, are reimbursed with the Basic Refund (50%) for any person living in the community. Patients with certain severe long-term diseases such as coronary heart disease (CHD), chronic hypertension or diabetes are entitled to a higher rate of refund (the Special Refund, 75 or 100%) and can be identified from a separate SII register. To be eligible for the Special Refund 1) the patient's condition must meet explicit, predefined criteria, 2) the patient must apply for the Special Refund using the necessary certificate completed by the treating physician and a SII's expert physician evaluates whether the criteria are met, and 3) the preparation dispensed must qualify for the Special Refund. Consequently, eligibility for a Special Refund for a certain disease can be used as a proxy for morbidity, although all patients potentially eligible do not apply for it. Overall, reimbursed drugs can be dispensed for a maximum period of three months for a single purchase.

There was no legal requirement for ethics committee approval since the intervention was directed at professionals, only unidentifiable patient data were used, and the patients were not contacted.

### Outcome measures

**Figure 2:**
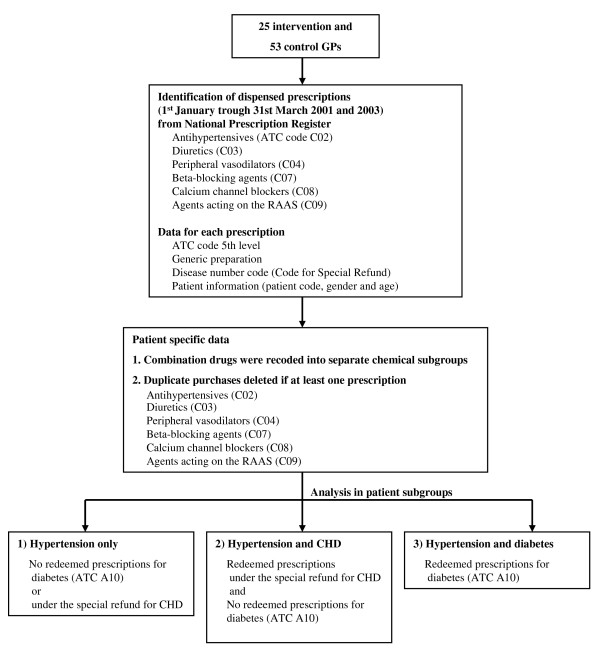
**The conversion of the prescription data to represent each patient's drugs**. Abbreviations: GP, general practitioner; ATC, Anatomical Therapeutic Chemical; RAAS, renin-angiotensin-aldosterone system; CHD, coronary heart disease.

The main outcomes were: 1) the proportion of patients treated with two or more concurrent antihypertensive drugs, 2) the proportion of patients with CHD treated with beta-blocking agents, 3) the proportion of patients with diabetes treated with RAAS, and 4) the proportion of patients with hypertension only treated with diuretics. The outcomes were measured during a three-month period before the first year of intervention, 1 January to 31 March 2001, and the respective period in 2003, after the intervention. The results are reported separately for hypertension patients with 1) hypertension only, 2) CHD, and 3) diabetes with or without CHD (Figure 2).

Reimbursed antihypertensive prescriptions issued by the participating physicians and dispensed from 1 January to 31 March 2001 and 2003 were retrieved from the Prescription Register, utilising doctors' personal codes. Irrespective of the refund category (Basic or Special Refund), all prescriptions for the following drugs were included: miscellaneous antihypertensives (ATC code C02), diuretics (C03), beta-blocking agents (C07), calcium channel blockers (CCBs) (C08) and agents acting on the renin-angiotensin-aldosterone system (RAAS) (C09). Dispensations were linked with a patient through the unique personal ID and converted to represent the drugs used by each patient (Figure 2). Consequently, the patient population, i.e. the denominator, for the study consisted of persons who were dispensed antihypertensive drugs in the first quarter of 2001 or 2003. We did not analyse previous use of antihypertensive drugs and therefore could not separate new users in the analysis. Due to the cross-sectional design, a patient's medication list could include continuously used drugs, new drugs added to the previous therapy as well as drugs discontinued during the specific three-month time window.

### Statistical analysis

Using logistic regression, adjusted odds ratios (ORs) for the use of specific antihypertensive agents (diuretics, beta-blocking agents, CCBs or RAAS) and the use of two or more concurrent antihypertensive drugs were estimated, comparing patients treated after the intervention with those treated before it. The 95% confidence intervals (95% CI) were calculated. Separate models were adapted for the intervention and control patients. Using data on both intervention and control patients, the statistical significance of the difference in the ORs between the groups was estimated by including an interaction term between time (after versus before) and group (intervention versus control) in the model. Because of the clustering of patients by physician, generalized linear mixed models were used in estimation, considering the physician as a random effect. All models were adjusted for the patients' age (continuous variable) and gender. The data was analysed using SPSS 15.0 for Windows and SAS 9.2.

The intervention was a pragmatic quality project and was based on the development needs of one organisation. Therefore the before and after design was chosen and power analysis was not performed *a priori*. To give the reader insight into the clinical significance of the results, confidence intervals for ORs are presented.

## Results

### Doctors

At baseline, the mean age of the intervention general practitioners (GPs) was 42 years and that of the control GPs 43 years. The number of female GPs was 22 (88%) and 33 (62%), respectively. The mean length of experience in primary care was 13 and 15 years. Participating GPs rated themselves as highly motivated to treat patients in accordance with the Current Care Guidelines (based on the Finnish school grading system from 4 to 10, median of 9 for both groups).

### Patients and the outcome measures

A total of 2,872 and 3,865 intervention patients and 7,066 and 8,693 control patients purchased antihypertensive agents in the data collection periods in 2001 and 2003, respectively (Table 2). In all patient cohorts the median age was 70 or more and approximately two-thirds were female. In all patient groups, the number of patients receiving two or more (max. four) concurrent antihypertensive drugs increased (Table 3). There was no difference in the change of likelihood of receiving two or more concurrent antihypertensive drugs versus monotherapy between intervention and control patients (p-values for intergroup comparisons > 0.05). The change for diabetes patients in the intervention group, compared to controls, was rather impressive (the percentage of patients receiving two or more concurrent antihypertensive agents +7.1% units, OR 1.33; 95% CI 0.99, 1.79, p = 0.06) but it did not reach statistical significance (p = 0.28).

**Table 2:** Characteristics of intervention and control patients in 2001 and 2003

	Intervention		Controls	
	**2001**	**2003**	**2001**	**2003**
	**(n = 2872)**	**(n = 3865)**	**(n = 7066)**	**(n = 8693)**

**Age, years, median (IQR)**	70 (62, 78)	71 (62, 78)	70 (61, 77)	71 (62, 78)
**Patients > 65 years**	1947 (67.8)	2654 (68.7)	4661 (66.0)	5915 (68.1)
**Females**	1823 (63.5)	2381 (61.6)	4315 (61.1)	5280 (60.8)
CDH*	320 (11.1)	440 (11.4)	950 (13.4)	1148 (13.2)
Diabetes*	302 (10.5)	456 (11.8)	612 (8.7)	828 (9.5)

Figures are number (percentage) unless otherwise stated.

Abbreviations: N, number; CHD, coronary heart disease; IQR, interquartile range.*Diagnosis of CHD was based on Special Refund code and diabetes on antidiabetic drug (ATC A10) purchases

**Table 3:** Number and proportions of patients who used two or more antihypertensive drugs concurrently and adjusted odds ratios for change

	Intervention				Controls				Intergroup comparison	
	**2001**	**2003**	Adjusted* OR (95% CI)	**p-value**	**2001**	**2003**	Adjusted* OR (95% CI)	**p-value**	Adjusted* OR (95% CI)	**p-value**
							
	**n (%)**				**n (%)**					

**Hypertension**										
	828 (36.8)	1164 (39.2)	1.12 (0.99, 1.25)	0.06	2119 (38.5)	2774 (41.3)	1.13 (1.05, 1.21)	0.002	0.99 (0.86, 1.13)	0.86
**CHD**										
	110 (34.4)	164 (37.3)	1.19 (0.87, 1.62)	0.23	292 (30.7)	389 (33.9)	1.10 (0.91, 1.33)	0.33	1.07 (0.75, 1.54)	0.70
**Diabetes**										
	144 (47.7)	250 (54.8)	1.33 (0.99, 1.79)	0.06	303 (49.5)	428 (51.7)	1.10 (0.89, 1.36)	0.38	1.22 (0.85, 1.75)	0.28

Prescription data was drawn from the national Prescription Register during a three-month period in 2001 and 2003.

Abbreviations: CHD, coronary heart disease; OR, Odds ratio; CI, confidence interval.

*Within-group analysis: Logistic regression, random effects model (physician as a random effect). Adjusted for patient's age and patient's sex. Reference group: patients with one antihypertensive drug.

**Intergroup analysis: Logistic regression, random effects model (physician as a random effect). A product term between time (after versus before) and group (intervention versus control) was added in the model. Adjusted for patient's age and patient's sex. Reference group: patients with one antihypertensive drug.

Apart from diuretics in most patient groups and calcium channel blockers in two groups, the number and proportion of patients receiving specific antihypertensive drugs increased for intervention and control patients (Table 4). The decrease of diuretics was significant only for control patients in the hypertension only group (-1.8% units, OR 0.92; 95% CI 0.85, 0.99, p = 0.02). There were no significant differences between the intervention and control patients in the change of use of specific antihypertensive agents (p-values for intergroup comparisons > 0.05). The use of beta-blocking agents increased in intervention patients with CHD (+6.1%-units, OR 1.39; 0.99, 1.96, p = 0.06). In the case of RAAS, the increase was mainly due to the increase in the use of angiotensin II receptor blockers (ARBs); for intervention patients with hypertension only the ORs were 1.61 (1.47, 1.78, p < 0.0001) and for control patients 1.58 (1.41, 1.78, p < 0.0001). For diabetic patients, the respective ORs were 2.21 (1.21, 4.04, p = 0.01) and 1.38 (0.95, 2.01, p = 0.09).

**Table 4:** Number and proportions of patients who used specific antihypertensive drugs and adjusted odds ratios for change

	Intervention				Controls				Intergroup comparison	
	**2001**	**2003**	Adjusted* OR (95% CI)	**p-value**	**2001**	**2003**	Adjusted* OR (95% CI)	**p-value**	Adjusted** OR (95% CI)	**p-value**
						
	**n (%)**				**n (%)**					

**Hypertension, N **	2250	2969			5504	6717				
Diuretics	886 (39.4)	1143 (38.5)	0.97 (0.86, 1.08)	0.56	2207 (40.1)	2575 (38.3)	0.92 (0.85, 0.99)	0.02	1.06 (0.92, 1.21)	0.44
Beta-blockers	982 (43.6)	1312 (44.2)	1.04 (0.93, 1.16)	0.50	2335 (42.4)	2890 (43.0)	1.03 (0.96, 1.12)	0.35	1.01 (0.88, 1.15)	0.93
CCBs	560 (24.9)	747 (25.2)	1.04 (0.92, 1.18)	0.55	1349 (24.3)	1682 (25.0)	1.04 (0.95, 1.13)	0.43	0.99 (0.84, 1.14)	1.00
RAAS	738 (32.8)	1108 (37.3)	1.19 (1.06, 1.34)	0.004	1966 (35.7)	2724 (40.6)	1.24 (1.15, 1.34)	<0.0001	0.96 (0.83, 1.10)	0.56
**CHD, N **	320	440			950	1148				
Diuretics	71 (22.2)	98 (22.3)	1.02 (0.71, 1.46)	0.92	171 (18.0)	229 (19.9)	1.05 (0.84, 1.33)	0.67	0.98 (0.64, 1.50)	0.92
Beta-blockers	235 (73.4)	350 (79.5)	1.39 (0.99, 1.96)	0.06	762 (80.2)	932 (81.2)	1.13 (0.90, 1.41)	0.29	1.25 (0.83, 1.88)	0.29
CCBs	103 (32.2)	113 (25.7)	0.75 (0.54, 1.03)	0.08	203 (21.4)	261 (22.7)	1.06 (0.86, 1.31)	0.59	0.70 (0.48, 1.03)	0.07
RAAS	43 (13.4)	86 (19.5)	1.57 (1.05, 2.34)	0.03	156 (16.4)	218 (19.0)	1.16 (0.92, 1.46)	0.20	1.34 (0.85, 2.12)	0.21
**Diabetes, N **	302	456			612	828				
Diuretics	115 (38.1)	184 (40.4)	1.10 (0.81, 1.49)	0.54	278 (45.4)	365 (44.1)	0.93 (0.75, 1.15)	0.49	1.19 (0.82, 1.72)	0.37
Beta-blockers	127 (42.1)	216 (47.4)	1.24 (0.93, 1.67)	0.15	275 (44.9)	379 (45.8)	1.03 (0.83, 1.28)	0.76	1.21 (0.84, 1.74)	0.31
CCBs	91 (30.1)	137 (30.0)	0.99 (0.72, 1.37)	0.96	148 (24.2)	223 (26.9)	1.13 (0.88, 1.45)	0.33	0.88 (0.59, 1.32)	0.54
RAAS	159 (52.6)	249 (54.6)	1.08 (0.80, 1.45)	0.64	284 (46.4)	427 (51.6)	1.27 (1.02, 1.57)	0.03	0.85 (0.59, 1.23)	0.39

Prescription data was drawn from the national Prescription Register during a three-month period in 2001 and 2003.

Abbreviations: CCBs, calcium channel blockers; RAAS, agents acting on renin-angiotensin-aldosterone system; CHD, coronary heart disease; OR, Odds ratio; CI, confidence interval.

*Within-group analysis: Logistic regression, random effects model (physician as a random effect). Adjusted for patient's age and patient's sex.

**Intergroup analysis: Logistic regression, random effects model (physician as a random effect). A product term between time (after versus before) and group (intervention versus control) was added in the model. Adjusted for patient's age and patient's sex.

## Discussion

Our findings suggest that the multifaceted comprehensive implementation of a hypertension guideline did not exert an impact on GPs' prescribing of antihypertensive drugs for drug-treated patients with hypertension, even though the participating GPs rated themselves as highly motivated to act according to the guidelines. Increased prescribing of beta-blocking agents for patients with CHD among intervention GPs and the increased prescribing of RAAS for diabetic patients among control GPs are more likely to reflect poor baseline adherence to the guideline than the effects of intervention. The observed ineffectiveness may be due to factors related to 1) intervention, 2) prescribing behaviour, or 3) study design and the measurements.

### Intervention

Planning, conducting and evaluating an extensive intervention is challenging, especially when a project is a quality programme aiming at changing healthcare processes rather than a pure clinical study. In extensive interventions, some aims may override others, especially if they are perceived to be more important by the participants [30]. In order to be adopted effectively, the aims must be explicit and repeatedly discussed with the participants. Our intervention focused on tailoring and implementing a local hypertension care path based on the national clinical guideline. The ultimate aim was to improve the treatment of hypertension patients by following the recommendations of the guideline, including drug therapy. For each session, the specific aims were highlighted and the topics were covered several times (Figure 1). The intervention was multifaceted, consisting of both lecture and small group education and the development of a local care path as well as audit and feedback on the division of tasks and treatment levels. Peer discussions and benchmarking were fundamental parts of the intervention. These methods should have had a small to modest impact on clinical practices, including prescribing [15].

### Prescribing behaviour

Prescribing is a complex behaviour simultaneously affected by several factors of varying intensity; the new information in the guidelines constitutes only one of these [31]. Among other things, both patients' and doctors' expectations and experiences may affect prescribing, as do the marketing efforts of the pharmaceutical industry. In our study, a decrease in use of diuretics for patients with hypertension only and an increase in the use of new ARBs were observed regardless of patients' co-morbidities and the fact that the guideline did not recommend ARBs as a first-line medication. Similarly, in the Netherlands in late 1990's, Grevings et al. observed that ARB prescriptions are not related to relevant co-morbidities [32].

Previous observations suggest that in order to achieve change in prescribing practices, personal feedback should be given to physicians [18,19]. In our intervention, GPs were informed of the data collection related to their prescribing and their consent to this was requested. However, since the intervention did not include personal feedback on prescribing, it may have been too imprecise to induce prescription-related change.

In other studies aiming at changing prescribing practices of antihypertensive drugs towards the guidelines, the improvement in proportions of target prescribing has been 5-13% units [21,33-35] and the controls have improved as well (2-6% units). In these studies the interventions have been more individualised. In our study, the prescribing of both the intervention and control physicians changed towards the guidelines. Firstly, we did not offer active interventions to the control GPs but they were informed about the measurements and permission was obtained from them. Secondly, the guideline was published and therefore available on the internet, and thirdly, the control GPs may have participated in local educational activities around the newly published guideline. Organisational traditions seem to modify physicians' prescription practices. Ohlsson and colleagues state that greater similarities seem to be found amongst prescription-related behaviour between physicians within the same health care unit than among those from different units [36]. In our intervention, peer discussions provided a good opportunity to reflect on prescription practices within the organisation, as well as its own practices against those of peers. Multidisciplinarity may have hindered the focusing of the discussion on prescribing although such a discussion is essential when improving organisational practices and task division.

### Study design and the measurements

As the intervention was a pragmatic quality project of one organisation, power analysis was not performed *a priori*. Type II error, under-powering the study, can not be ruled out. Therefore, it is possible that we failed to reach statistically significant changes even though the intervention actually was effective. For example, two changes in the intervention group - the percentage of patients with diabetes receiving two or more concurrent antihypertensive agents increased by 7.1% units (OR 1.33; 95% CI 0.99, 1.79, p = 0.06) and the percentage of patients with CHD using beta-blocking agents increased by 6.1% units (OR 1.39; 0.99, 1.96, p = 0.06) - nearly reached statistical significance.

The intervention GPs, the participants in the quality project, were voluntary. Similarly the controls were invited on a voluntary basis, in order to reduce selection bias. This self-selection of active GPs may reduce the generalizability of the results to the general GP population. Furthermore voluntary participants in studies may be active in the adoption of new information and therefore correspondingly active in bringing about change.

We found no differences in measured patient characteristics between intervention and control patients or within either group before and after the intervention. The case-mix in terms of age, gender and measured morbidities probably did not affect the results as we stratified the patients by co-morbidities. However, we did not control for other patient-related factors such as socioeconomic status or use of other health care services.

In Finland, the Special Refund for diabetes is often delayed [37]: we therefore chose purchases of antidiabetic drugs as the definition of the disease. Diagnosis of CHD based on a Special Refund is specific, but may not be sensitive. Consequently, patients with CHD may have been misclassified into the hypertension only group. This, in turn, may have led to an overestimation of the use of beta-blockers as hypertension medication. Previous studies, however, have shown that in Finland, beta-blocking agents are preferred to other antihypertensives [9,13,38].

In the following, we will focus on measuring the impact of an intervention accurately in the context of pharmacy claims databases and give specific consideration to our outcome measures in the light of the issues raised by Maclure et al. (2006) in their review of measuring prescription-related improvements in the primary care setting [39].

We measured change in prevalence of use of various antihypertensive medications. When measuring prescribing, the denominator, i.e. the definition of the patient group in which the outcome is intended to be measured, is critical. As Maclure and co-workers stated, every patient in the denominator should be at risk of entering the numerator [39]. Therefore, new patients visiting a physician - i.e. incident users of any antihypertensives, those switching from one therapeutic class to another and those adding a drug to their previous medication - would represent a more valid patient group for measuring changes in prescribing. In our study the denominator consisted of both new and previous users of any antihypertensive drugs - potentially leading to the dilution of the intervention's observed effect. One explanation is that, from the clinical point of view, there is no need for change if the drug is effective and well tolerated, even if the treatment was not recommended in the guideline as a first line medication. On the other hand, because switching was not analysed we may have overestimated the concurrent use of various drugs. Our analysis of ACEI and ARBs as a single group (RAAS), where switching between the drugs is usual, diminishes this bias.

We only had data on purchased prescriptions reflecting both doctors' and patients' actions. Combining patient records and claims data would provide more valid information on doctors' practices. Furthermore, we retrieved claims for the prescriptions written by the study GPs only and may have overestimated the number of patients on monotherapy in case other physicians prescribed hypertension medication to the same patients. Finally, the duration of the follow-up and the timing of the measurements have an influence on whether an intervention's outcomes and impact can be detected [39]. In Finland, a prescription is valid for one year from the date it is issued; thus, patients with chronic conditions and a good treatment balance need to visit a doctor or renew their prescriptions at least once a year. For this reason, our three-month time window may reflect prescribing during the preceding year. A longer follow-up would be needed to detect changes, especially if only new patients were included in the analysis.

### Implications

The latest update of the Current Care Hypertension Guideline published in 2009 included several new recommendations [40]. A combination of RAAS and CCB was recommended over one of RAAS and diuretics, especially for high-risk patients, and combinations with beta-blockers were discouraged. Concern has been expressed on how the new recommendations will be translated into clinical practice, especially in a country characterised by the frequent use of beta-blocking agents.

Practical tools, such as quality indicators, could facilitate guideline implementation. Various indicators have been used to measure the quality of prescribing and monitor change [41-45]. However, the development processes of various indicators may not be explicit [46]. Measures should be focused, valid in every sense (content, face, concurrent, construct) and feasible. The evidence base underlying prescribing indicators should be clearly described; for example, in order to have strong content validity, indicators should be based on up-to-date guidelines [47,48]. They should be developed with three aims in mind: the evaluation of clinical practices at the personal and organisational level as well as providing national benchmarking data. Comprehensive guideline implementation and health care management could be strengthened if, when using indicators, areas with the potential for improvement were recognised prior to conducting implementation and quality improvement activities. Researchers would also benefit; evidence on changing prescribing and implementation would be easier to collect. Furthermore, claims databases and electronic patient records should support data collection on clinical practices and patient outcomes.

## Conclusions

A multifaceted guideline implementation intervention does not necessarily lead to significant changes in prescribing performance. Detected intra-group changes toward the guideline seem more likely to reflect poor baseline adherence than the effects of intervention. Lack of personal feedback on prescribing may have been one reason for ineffectiveness. Future research in this area would benefit from larger samples to avoid the danger of under-powering a study and to overlook the positive effects of multifaceted interventions.

## Competing interests

The authors declare that they have no competing interests.

## Authors' contributions

RS: participated in the design of the study, performed the statistical analysis and wrote the first draft of the manuscript. AHS participated in the design of the study, participated in the manuscript drafting and revised the manuscript critically. MJK advised and participated in the statistical analysis and revised the manuscript critically. EK: participated in the design of the study and revised the manuscript critically. All authors read and approved the final manuscript.

## Pre-publication history

The pre-publication history for this paper can be accessed here:

http://www.biomedcentral.com/1471-2296/12/87/prepub
